# Association of cholinergic integrity measured by [^18^F]‐fluoroethoxybenzovesamicol with age and amyloid burden in adults with Down syndrome

**DOI:** 10.1002/alz70856_099363

**Published:** 2025-12-24

**Authors:** Jason K Russell, Alexander C Conley, Brian Boyd, John Patrick Begnoche, Rachel Schlossberg, Allison Stranick, Adam J Rosenberg, Lealani Mae Y. Acosta, Dann Martin, Yasmeen Neal, Prabesh Kanel, Roger L. Albin, Michael S. Rafii, Julie Dumas, Paul A Newhouse

**Affiliations:** ^1^ Center for Cognitive Medicine, Vanderbilt University Medical Center, Nashville, TN, USA; ^2^ Center for Cognitive Medicine, Vanderbilt University Medical Center, Nashville, TN, USA, Nashville, TN, USA; ^3^ Vanderbilt University Medical Center, Nashville, TN, USA; ^4^ University of Michigan, Ann Arbor, MI, USA; ^5^ Alzheimer's Therapeutic Research Institute, Keck School of Medicine, University of Southern California, San Diego, CA, USA; ^6^ University of Vermont, Burlington, VT, USA

## Abstract

**Background:**

Adults with Down syndrome (DS) are at increased risk for Alzheimer's disease (AD) due to *APP* triplication. Cholinergic system degeneration underlies many AD‐related cognitive deficits, but cholinergic integrity in adults with DS is not well defined. We use [^18^F]‐fluoroethoxybenzovesamicol ([^18^F]‐FEOBV) PET imaging to measure regional cholinergic terminal density in adults with DS compared to age‐ and amyloid‐matched neurotypical controls.

**Method:**

Sixteen non‐demented adults with DS were recruited from the Trial Ready Cohort – Down Syndrome and *de novo* to a cholinergic study (8 female, 35.5 years). Twenty (10 female, 35.5 years) age‐matched and fifteen (15 female, 61.5 years) amyloid‐matched participants were recruited for cognitively normal control groups. All subjects received [^18^F]‐FEOBV PET and MRI scans, fifteen adults with DS and amyloid‐matched individuals received amyloid scans ([^11^C]‐PiB or [^18^F]‐Florbetabir). Following normalization to MNI‐space and partial volume correction, a voxelwise t‐test compared [^18^F]‐FEOBV uptake between adults with DS and age‐matched controls. General linear models assessed the relationship between [^18^F]‐FEOBV uptake, age, or amyloid in adults with DS. Group x age and group x amyloid interaction analyses assessed if [^18^F]‐FEOBV relationships differed between adults with DS and control participants.

**Result:**

Adults with DS exhibited increased [^18^F]‐FEOBV uptake in the cerebellum, brainstem, thalamus, and cortical regions compared to age‐matched neurotypical controls (*p* < 0.001). They also exhibited lower uptake in cortical clusters among older participants, along with a significant age x group interaction revealing a greater reduction in adults with DS compared to controls (*p* < 0.005). Additionally, adults with DS displayed lower [^18^F]‐FEOBV uptake related to higher amyloid accumulation, showing a more significant decline compared to amyloid‐matched controls (*p* < 0.005). All analyses had a minimum cluster size of 50.

**Conclusion:**

These data indicate an upregulation of cholinergic terminal markers in adults with DS by early adulthood, suggesting greater subsequent declines due to age and amyloid pathology relative to controls. Significant age and amyloid associations overlap in some regions, with more clusters exhibiting significant age‐associated effects. This result suggests that declining regional [^18^F]‐FEOBV uptake in adults with DS is influenced by both Alzheimer's‐related pathology and aging, each playing distinct yet overlapping roles.

